# Epithéliomas basocellulaires de la face: prise en charge chirurgicale, à propos de 45 cas et revue de la literature

**DOI:** 10.11604/pamj.2014.19.80.5187

**Published:** 2014-09-25

**Authors:** Mohamed Amine Ennouhi, Abdenacer Moussaoui

**Affiliations:** 1 Service de Chirurgie Plastique, Réparatrice et des Brûlés, Hôpital Militaire d'Instruction Mohammed V, Rabat, Maroc

**Keywords:** Epithélioma basocellulaire, face, chirurgie, anatomopathologie, Basal cell basal cell epithelioma, face, surgery, pathology

## Abstract

L’épithélioma basocellulaire est de loin la tumeur épithéliale maligne la plus répandue. L'atteinte faciale représente plus de 65% des cas et constitue un facteur de risque de récidive. L'objectif de notre travail est de rappeler les principes et modalités du traitement chirurgical. Sur une période de douze mois, nous avons pris en charge quarante-cinq patients atteints de carcinomes basocellulaires de la face. Le traitement chirurgical comprend deux volets: -carcinologique: emportant la tumeur et une marge de tissu sain; -et une chirurgie réparatrice faisant appel à la suture cutanée directe; greffes ou lambeaux loco -régionaux. L'examen histologique systématique des pièces opératoires permet la confirmation du diagnostic, le typage histologique et l'appréciation de la qualité de l'exérèse chirurgicale. Les résultats esthétiques sont jugés satisfaisants. Quant aux résultats carcinologiques, nous déplorons quatre récidives. Le traitement chirurgical des épithéliomas basocellulaires est le seul garant de la guérison. Au niveau de la face, il faut trouver le meilleur compromis entre impératifs carcinologiques et esthétiques. L'amélioration des résultats passe par: la prévention, le dépistage précoce des lésions, la collaboration étroite des anatomo-pathologistes et la création de comités de concertation pluri -disciplinaire pour la prise en charge des cas difficiles.

## Introduction

L’épithélioma ou carcinome basocellulaire est de loin la tumeur épithéliale maligne la plus répandue; et représente 75% des cancers cutanés non mélaniques [1]. L'atteinte faciale représente entre 65% et 85% des cas [2, 3] et constitue un facteur de risque de récidive [4, 5].la chirurgie est le traitement de référence de cette lésion; Elle permet un taux élevé de guérison, notamment par le contrôle histologique des marges. L'objectif de notre travail est de rappeler les principes et modalités du traitement chirurgical.

## Méthodes

Sur une période de 12 mois, nous avons pris en charge 45 patients atteints de carcinomes basocellulaires de la face; dont deux récidives et un cas en poussée évolutive. Le traitement chirurgical; conduit sous anesthésie locale, locorégionale ou générale comprend deux volets: carcinologique: l'exérèse tumorale emporte en périphérie une marge de sécurité variant de 3 à 10 mm. En profondeur, elle emporte l'hypoderme jusqu’à la première barrière anatomique exclue. Les pièces opératoires sont envoyées systématiquement au laboratoire d'anatomie pathologique. L'examen histologique, réalisé après fixation des pièces, permet: la confirmation du diagnostic, le typage histologique et l'appréciation de la qualité de l'exérèse chirurgicale. Pour des raisons techniques, liées à un effectif restreint du laboratoire d'anatomo-pathologie, aucun examen extemporané n'a pu être réalisé. -une chirurgie réparatrice faisant appel selon le besoin à une ou plusieurs techniques de réparation cutanée: cicatrisation dirigée, suture cutanée directe, greffes, lambeaux... Les patients sont revus à 3 mois, 6 mois et 1 an; puis tous les ans.

## Résultats

**Figure 1:**
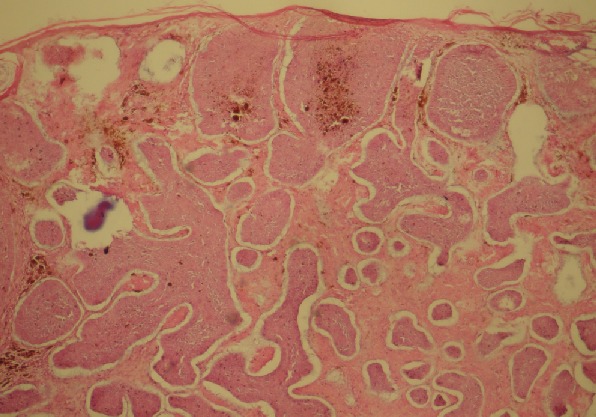
Aspect histologique d'un épithélioma basocellulaire nodulaire (coloration H.E, grossissement ×10)

**Figure 2:**
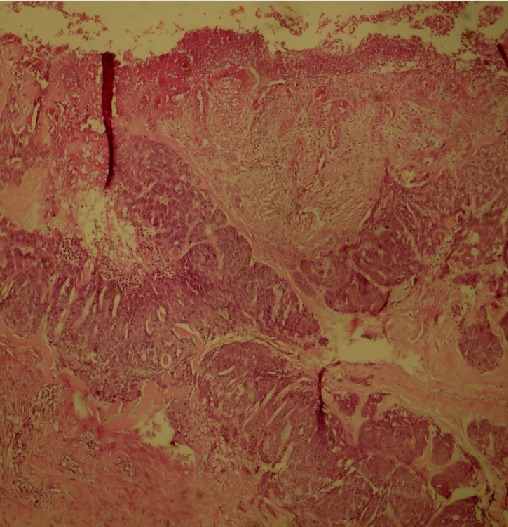
Aspect histologique d'un épithélioma basocellulaire infiltrant (coloration H.E, grossissement ×10)

**Age:** L’âge moyen de nos patients est de 66 ans. Les extrêmes sont de 37 et 80 ans. **Sexe:** on note une prédominance masculine, avec un sexe ratio = 2. **Taille tumorale:** elle varie entre 6 et 32mm; la moyenne est de 16mm. **Localisation:** la localisation nasale est la plus fréquente (53% des cas), suivie de la région orbito-palpébrale (25% des cas). La lésion siège aux étages moyen et supérieur de la face dans 93% des cas. **Type histologique:** nodulaire (Figure 1) dans 53% des cas. *infiltrant (Figure 2) dans 40% des cas. *sclérodermiforme dans 6% des cas. **Marge d'exérèse:** macroscopiquement, elle varie entre 3 et 10mm. La qualité de l'exérèse tumorale est jugée satisfaisante dans 39 cas; dans 6 cas elle a été jugée incomplète, ce qui a motivé une reprise du lit tumoral et confirmation du caractère carcinologique de l'exérèse avant couverture dans 5 cas.

**Traitement:** la chirurgie s'est déroulée en deux temps dans 30 cas, la couverture a été réalisée après confirmation du caractère complet de l'exérèse tumorale par l'examen histologique définitif. La reconstruction a fait appel à: la cicatrisation dirigée: 3 cas; la suture directe: 3 cas; Greffe de peau totale: 13 cas; lambeaux loco – régionaux: 27 cas. Les résultats esthétiques sont jugés sur la qualité et l'emplacement des cicatrices, le respect des sous-unités esthétiques et l'intégration du tissu de recouvrement dans la zone receveuse. Dans l'ensemble, ils sont satisfaisants. Quant aux résultats carcinologiques, nous disposons d'un recul moyen de trois ans, avec des extrêmes de 18 et 72 mois. Nous déplorons quatre récidives dont trois sont locales, la quatrième est régionale sous forme de métastases ganglionnaires, le patient est malheureusement décédé quelques mois plus tard. Le délai de survenue des récidives est de 18 mois en moyenne. Les données des quatre patients ayant récidivé sont récapitulées dans le Tableau 1.

**Tableau 1:** Récapitulatif des données cliniques, histologiques et thérapeutiques des patients ayant récidivé

Patient	Age	Taille tumeur primitive	Siège	Type histologique	Traitement de la tumeur primitive	Marge d'exérèse (en mm)	Délai de récidive (en mois)	Traitement de la récidive
Patient 1	73 ans	15mm	Canthus interne	infiltrant	Exérèse - greffe de peau	1	12	Exerèse - couverture par lambeau Frontal + Canthpexie interne
Patient 2	37 ans	10mm	Canthus interne	Infiltrant	Exérèse - greffe de peau	3	30	Exerèse - couverture par lambeau Frontal
Patient 3	59 ans	20mm	Sillon naso-génien	infiltrant	Exérèse - greffe de peau	3	24	Exérèse suivie de couverture par lambeau Frontal +lambeau Temporo-jugal
Patient 4	57 ans	11mm	Lèvre inférieure	Scléro-dermiforme	Exérèse-suture	3	6	Exérèse+ curage cervical fonctionnel bilatéral + couverture par lambeaux hétéro labiaux et lambeau cervical Suivis de radiothérapie

## Discussion

**Figure 3:**
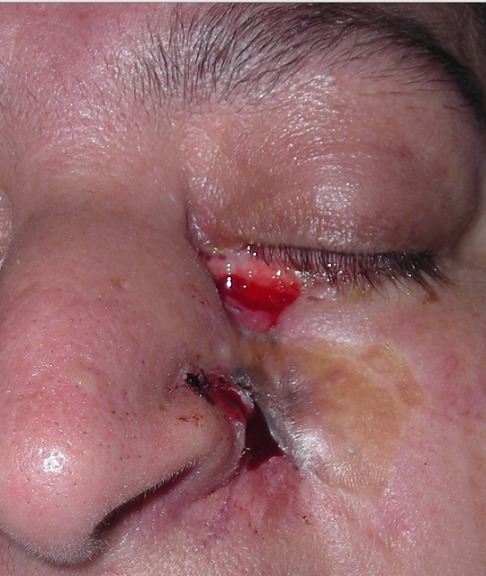
Cas clinique illustrant une récidive d'un CBC et l'importance de l'envahissement local et le préjudice fonctionnel et esthétique qui en résulte; basocellulaire récidivant du sillon naso-génien avec envahissement du canthus interne et de la paupière inférieure

**Figure 4:**
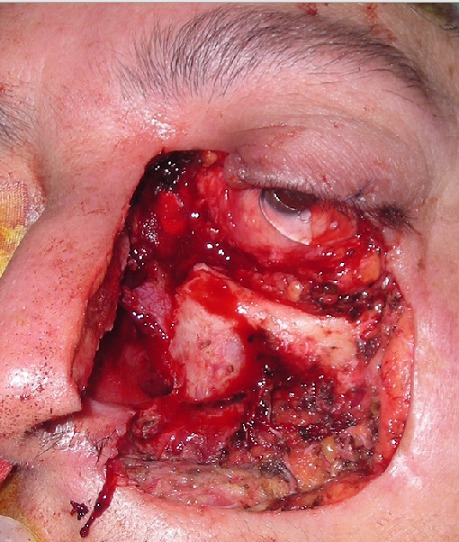
Cas clinique illustrant une récidive d'un CBC et l'importance de l'envahissement local et le préjudice fonctionnel et esthétique qui en résulte; exérèse emportant la paupière inférieure, le canthus interne, la paroi latérale du nez, l'aile du nez et la joue

**Figure 5:**
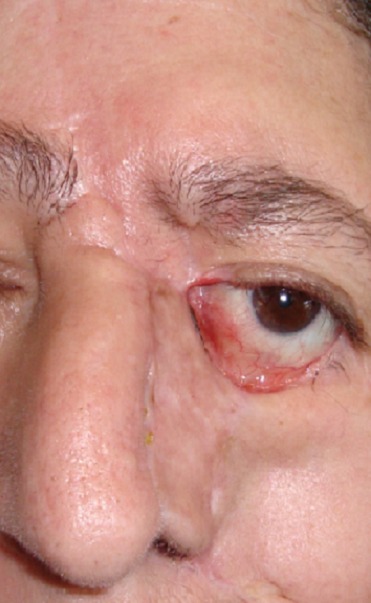
Cas clinique illustrant une récidive d'un CBC et l'importance de l'envahissement local et le préjudice fonctionnel et esthétique qui en résulte; couverture par lambeau temporo-jugal d'avancement - rotation + lambeau frontal retourné et greffé Réfection palpébrale inférieure prévue ultérieurement

L’épithélioma ou carcinome basocellulaire (CBC) représente un tiers des cancers dans les pays occidentaux et 80% des cancers cutanés en dehors du mélanome [6]. Son incidence croit rapidement [1, 7]. En France, elle serait de 70 pour 100000 habitants [8]. Au Maroc, il constitue 61% des cancers cutanés, et siège dans 80% des cas au niveau de la région cervico-faciale [6]. Sous prétexte que l’évolution est lente et locale, le carcinome basocellulaire est pris pour une tumeur à malignité réduite. Or, certaines formes peuvent être très mutilantes d'autant plus qu'au niveau de la face, L’épaisseur des parties molles recouvrant le squelette est faible et le danger d'extension dans les zones de fusion des bourgeons embryonnaires est important [1, 9, 10]. Par conséquent, le préjudice fonctionnel et esthétique peut être considérable (Figure 3, Figure 4 et Figure 5).

Le traitement chirurgical des épithéliomas basocellulaires est le seul garant de la guérison [11]. Toute ablation tumorale maligne ne doit être guidée que par les impératifs carcinologiques; les éventuelles difficultés de réparation des pertes de substance n'ont pas à interférer dans les modalités et la conduite de l'exérèse [12], sauf si l'on décide de prendre un risque pour respecter un organe noble [13]. Si la majorité des auteurs s'accordent sur l'importance d'une exérèse chirurgicale complète du CBC, ils ne s'accordent pas pour autant sur la définition d'une marge de sécurité « standard ». Ainsi, Pour une même lésion, située au même endroit, les marges d'exérèse varient en fonction des opérateurs et des services [14]. Compte tenu de la multiplicité des facteurs pouvant influer sur la décision thérapeutique, l'ANAES recommande dans son rapport de 2004 [4] de classer les patients atteints de CBC en 3 groupes pronostiques: mauvais, intermédiaire et bon. Cette classification basée sur des critères cliniques et histologiques permet de recommander une prise en charge diagnostique et thérapeutique adaptée aux critères pronostiques identifiés. Pour ce qui est des critères cliniques: *Sont de mauvais pronostic: -la localisation nasale et péri-orificielle [4, 14, 15]; -la taille supérieure à 10mm. -les formes mal limitées et récidivantes. *Les critères de bon pronostic sont: -la localisation extra céphalique (tronc et membres); -les formes superficielles. Les critères histologiques de mauvais pronostic sont les basocellulaires sclérodermiforme, infiltrant et les formes métatypiques. En cas d'association, le pronostic global dépend de la composante de plus mauvais pronostic.

Dans notre série: 27 patients sont classés de mauvais pronostic, soit 60% des cas. 15 patients sont de pronostic intermédiaire. Et trois sont de bon pronostic. Après avoir identifié les groupes pronostiques, L'ANAES [4] recommande de respecter une marge de 3mm dans les formes de bon pronostic; et d'aller jusqu’à 10mm, dans les formes de mauvais pronostic. L’évaluation de l'efficacité du traitement chirurgical des CBC repose sur un critère principal: le taux de récidive [16]. Ce dernier est en étroite relation avec la qualité de l'exérèse [14]. Sur une durée de cinq ans, Pascal et al. [17] ont étudié la survenue de récidive en fonction de la distance plan d'exérèse-tumeur, leur étude incluait 143 carcinomes basocellulaires. Le taux de récidive était de 1,2% lorsque la distance plan d'exérèse-tumeur était supérieure à 5mm. En dessous, le taux de récidive était dix fois plus important, soit 12%. Lorsque la tumeur arrivait au contact des berges, le taux de récidive était de 33% [14, 17]. Ce dernier résultat est confirmé par d'autres études [18–21] qui retrouvent entre 21 et 41% de récidive quand l'exérèse est histologiquement incomplète. Selon Boulinguez et al. 24% des CBC incomplètement excisés récidiveraient sous une forme plus agressive [22, 23].

Dans notre série, pour les quatre CBC ayant récidivés, les marges d'exérèse calculées par l'anatomopathologiste ont été comprises entre 1 et 3 mm. Malheureusement, les paramètres de mauvais pronostic relevés dans les quatre cas (Tableau 1) n'ont pas été suffisamment pris en compte et l'attitude chirurgicale aurait due être plus radicale. Dans son étude portant sur 674 basocellulaires, STAUB et al. [14], rapporte plus de 95% d'absence de récidive à cinq ans en utilisant des marges d'exérèse moyennes de 4 mm pour les carcinomes basocellulaires non sclérodermiforme, et de 8 à 10 mm pour les sclérodermiformes et les lésions périorificielles. Mais, dans certaines localisations (à proximité de l’œil par exemple), se pose parfois le dilemme entre le respect strict des marges de sécurité et la préservation de la fonction. Dans de telles circonstances, la chirurgie micrographique de Mohs (CMM) peut être une alternative offrant une guérison maximale tout en sacrifiant un minimum de tissu sain pour arriver à un résultat cosmétique et fonctionnel satisfaisant [11]. Technique décrite pour la première fois par Frederic Mohs en 1941 [24], elle est largement répandue aux USA. Le principe de cette chirurgie est d’étudier 100% des marges latérales et en profondeur, contrairement aux techniques habituelles qui n'analysent que 1% des marges d'exérèse [4, 11, 25]. La CMM est la technique pour laquelle les taux de récidive rapportés dans la littérature sont les plus faibles, particulièrement pour le traitement des basocellulaires de mauvais pronostic [4, 16, 26].

Dans une étude rétrospective, portant sur 587 basocellulaires agressifs de la face traités par CMM, J.PAOLI et al. [27] rapportent des taux de récidive à 5ans de 2,1% pour les CBC primitifs et 5,2% pour les CBC récidivants. Pour d'autres auteurs [11, 28, 29] ces taux varient entre 4% et 6,5% pour les CBC primitifs et entre 6% et 10% pour les formes récidivantes. K. Mosterd et al. [30] ont publié en 2008 les résultats d'une étude prospective randomisée comparant la chirurgie micrographique de Mohs à l'excision chirurgicale des basocellulaires primitifs et récidivants de la face. Les taux de récidive à 5 ans des CBC primitifs et récidivants sont respectivement de 4,1% et 12,1% après chirurgie standard, et de 2,5% et 2,4% après CMM. Les auteurs de cette étude concluent à la supériorité de la CMM dans le groupe des CBC récidivants et à l'absence de différence significative entre les deux méthodes dans le groupe des CBC primitifs de la face. Il conviendrait donc de limiter les indications de la CMM aux CBC à haut risque de récidive [23] tels que les CBC récidivants, les localisations péri-orificielles et les CBC infiltrant et sclérodermiforme. Quoi que les métastases ganglionnaires du carcinomebasocellulaire soient exceptionnelles, la présence d'adénopathies dans le territoire de drainage de la tumeur peut être en rapport avec des métastases ganglionnaires. Cette éventualité justifie à notre sens la réalisation d'un curage ganglionnaire sélectif afin d'asseoir le diagnostic précis de ces adénopathies.

**Figure 6:**
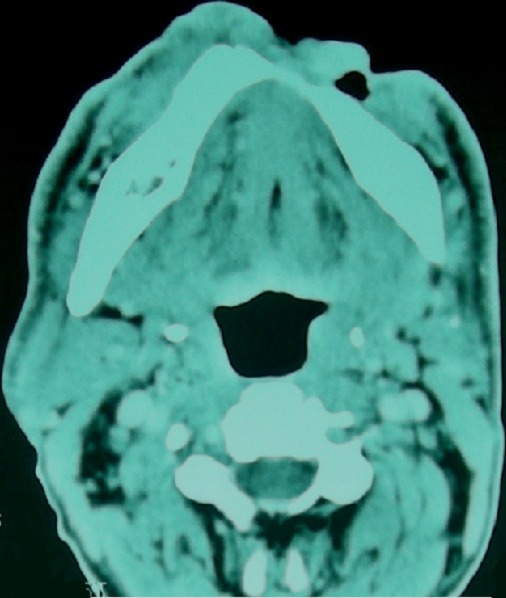
TDM cervico-faciale d'un épithélioma basocellulaire récidivant de la lèvre inférieure et de la région mentonnière avec métastase ganglionnaire régionale; processus ulcéro-bourgeonnant des parties molles de la région mentonnière

**Figure 7:**
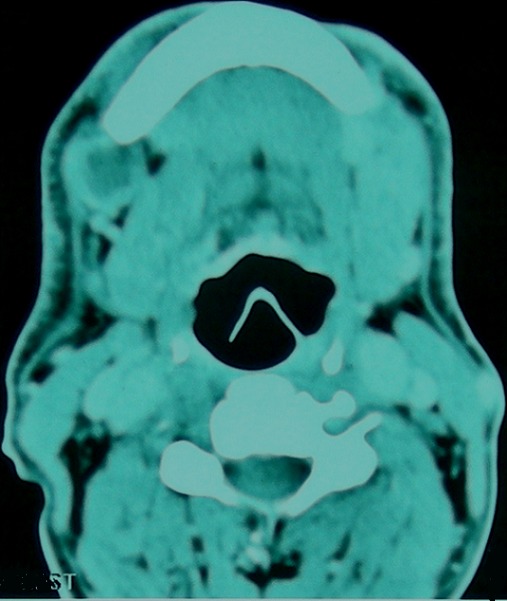
TDM cervico-faciale d'un épithélioma basocellulaire récidivant de la lèvre inférieure et de la région mentonnière avec métastase ganglionnaire régionale; adénopathie sous-angulo-mandibulaire droite à centre nécrotique

Dans notre série, nous avons colligé un cas d’épithélioma basocellulaire sclérodermiforme de la lèvre inférieure et de la région mentonnière (Figure 6) traité initialement par des exérèses itératives incomplètes. L’évolution a été marquée par l'apparition d'adénopathies cervicales bilatérales intéressant les groupes Ib droit et gauche (Figure 7). On a réalisé un curage sélectif bilatéral. L’étude histologique des ganglions prélevés a porté le diagnostic de métastase de carcinome basocellulaire. Le patient a été traité par radiothérapie et est décédé, malheureusement, quelque mois plus tard. Nous estimons que le caractère incomplet et insuffisant du traitement chirurgical initial serait à l'origine de l'essaimage tumoral. Enfin, nous mettons l'accent sur la nécessité d'un traitement chirurgical bien réfléchi, prenant en considération les différents facteurs pronostiques et soulignons l'intérêt d'un examen anatomopathologique rigoureux des pièces opératoires.

## Conclusion

Le carcinome basocellulaire est une tumeur fréquente sous nos cieux. L'atteinte du visage, étant un élément de mauvais pronostique de récidive, expose à des préjudices esthétiques et fonctionnels lourds. L'amélioration des résultats passe par: la prévention (protection anti-soleil, information de la population); le dépistage précoce des lésions, offrant ainsi au patient des chances de guérison plus importantes avec le minimum de séquelles; le développement des plateaux techniques des centres d'anatomopathologie; la création de comités de concertation pluri-disciplinaire réunissant: dermatologues -chirurgiens plasticiens, anatomopathologistes et cancérologues pour la prise en charges des cas difficiles.
